# Psychosocial Functioning and Intelligence Both Partly Explain Socioeconomic Inequalities in Premature Death. A Population-Based Male Cohort Study

**DOI:** 10.1371/journal.pone.0082031

**Published:** 2013-12-11

**Authors:** Daniel Falkstedt, Kimmo Sorjonen, Tomas Hemmingsson, Ian J. Deary, Bo Melin

**Affiliations:** 1 Department of Public Health Sciences, Karolinska Institutet, Stockholm, Sweden; 2 Division of Psychology, Department of Clinical Neuroscience, Karolinska Institutet, Stockholm, Sweden; 3 Institute of Environmental Medicine, Karolinska Institutet, Stockholm, Sweden; 4 Centre for Social Research on Alcohol and Drugs, Stockholm University, Stockholm, Sweden; 5 Centre for Cognitive Ageing and Cognitive Epidemiology, Department of Psychology, The University of Edinburgh, Edinburgh, United Kingdom; University of California, San Francisco, United States of America

## Abstract

**Objective:**

The possible contributions of psychosocial functioning and intelligence differences to socioeconomic status (SES)-related inequalities in premature death were investigated. None of the previous studies focusing on inequalities in mortality has included measures of both psychosocial functioning and intelligence.

**Methods:**

The study was based on a cohort of 49 321 men born 1949–1951 from the general community in Sweden. Data on psychosocial functioning and intelligence from military conscription at ∼18 years of age were linked with register data on education, occupational class, and income at 35–39 years of age. Psychosocial functioning was rated by psychologists as a summary measure of differences in level of activity, power of initiative, independence, and emotional stability. Intelligence was measured through a multidimensional test. Causes of death between 40 and 57 years of age were followed in registers.

**Results:**

The estimated inequalities in all-cause mortality by education and occupational class were attenuated with 32% (95% confidence interval: 20–45%) and 41% (29–52%) after adjustments for individual psychological differences; both psychosocial functioning and intelligence contributed to account for the inequalities. The inequalities in cardiovascular and injury mortality were attenuated by as much as 51% (24–76%) and 52% (35–68%) after the same adjustments, and the inequalities in alcohol-related mortality were attenuated by up to 33% (8–59%). Less of the inequalities were accounted for when those were measured by level of income, with which intelligence had a weaker correlation. The small SES-related inequalities in cancer mortality were not attenuated by adjustment for intelligence.

**Conclusions:**

Differences in psychosocial functioning and intelligence might both contribute to the explanation of observed SES-related inequalities in premature death, but the magnitude of their contributions likely varies with measure of socioeconomic status and cause of death. Both psychosocial functioning and intelligence should be considered in future studies.

## Introduction

From adulthood, individuals exhibit differences in intelligence (IQ) and personality traits that are relatively stable over the life course [1]. By being associated both with attainment of socioeconomic status (SES) [2], [3], [4] and with health and longevity [1], [5], such individual differences may contribute to explain persistent inequalities in mortality between SES levels. That is, SES may be confounded by intelligence/personality traits in its association with mortality.

Gottfredson's hypothesis that differences in intelligence are “the Epidemiologists' elusive ‘fundamental cause’ of social class inequalities in health” [6] spurred a number of empirical studies, with conflicting results. Whereas analyses of data from the Whitehall II study [7], from the U.S. National Longitudinal Survey of Youth study [8], and from the Malmö Longitudinal study in Sweden [9] were interpreted as not supporting intelligence as an important explanation, analyses of Scottish studies [10], [11], the Vietnam Experience study [12], and a very large study of young Swedish men [13] provided some supportive evidence. The possible importance of personality traits, i.e., people's tendencies to behave, think, and feel in certain ways [14], [15], has recently been supported in analyses of data from the French GAZEL study [16] and the Midlife Development in the United States (MIDUS) cohort [17]. In both studies, associations between SES and mortality were attenuated after adjustment for measures of personality. However, in the Vietnam Experience study a measure of the neuroticism dimension of personality was not found to contribute to the association between income and mortality [18], for which IQ proved to be important. None of the studies with a primary focus on explaining SES-related inequalities in mortality included measures of both intelligence and personality characteristics, however. Yet, it is possible that these factors are to some extent overlapping explanations of observed inequalities in mortality [19].

Broadly, hypotheses about the associations of intelligence and personality differences with causes of death, i.e. associations that may confound observed SES-related inequalities in mortality, posit that individual psychological differences, on the one hand, reflect varying ability among individuals to deal with long-term risks of disease and death; on the other hand, they might be associated with hazardous behaviors or selection into the poorer conditions of lower SES groups [20], [21]. Indicators of attained SES and health-related behaviors have been demonstrated to account for associations between IQ and causes of death in previous studies [5] but have not explained associations between personality characteristics and causes of death to the same extent [1]. Attained SES is however related both to personality traits and to IQ [2], [3], and in a previous study we found that IQ was associated with SES in terms of education and occupational class, whereas aspects of personality may relate more to SES measured by income level [22].

The purpose of the present study was to examine the question of whether individual differences in personality characteristics and intelligence associated with the attainment of SES might both be factors that contribute to observed SES-related inequalities in mortality. Analyses were carried out on a large cohort with prospective data. IQ and psychosocial functioning were measured in late adolescence, SES in early middle age, and major causes of death during follow-up between 40 and 57 years of age. SES was measured by level of education and occupational class, as well as by level of income, which are commonly used, but not identical, indicators of SES [23]; it is possible that they are affected by intelligence and psychosocial functioning in partly different ways [22]. Psychosocial functioning and IQ have not previously been investigated in this cohort as explanations of SES inequalities in mortality, although IQ-mortality associations have been reported previously [24], [25].

## Methods

### Study population

The study was based on a cohort of Swedish males who were conscripted into compulsory military service in 1969/1970. Only 2–3% of all Swedish men were exempted from conscription at this time, in most cases due to severe handicaps or congenital disorders. Approximately 98% were born 1949–1951. The men born 1949–1951 were 49 321 in total, were all aged 18–20 years at conscription, and were all between 40 and 57 years of age approximately during the follow-up period from 1991 to 2008. During conscription, at any of seven regional conscription centres, each conscript underwent a series of tests of physical and mental health status, psychosocial functioning, and intelligence; full medical examinations were carried out; and self-administered questionnaires on family, social background, behavior and adjustment, and health and substance use were completed.

### Ethics statement

The study was approved by the Stockholm Regional Ethical Review Board in Sweden (Dnr 2010/604-32). Due to the character of the data base and the anonymization of all data, the Review Board waived the normal requirement for written consent.

### Measures of intelligence and psychosocial functioning

Psychometric assessment of intelligence was conducted during conscription through the use of four subtests, measuring verbal ability, logical-inductive ability, visuospatial ability, and technical comprehension. Results were converted into normally-distributed standard-nine (stanine) scales for each subtest, with scores 1 to 9, and the scales were then combined and transformed onto a new stanine scale as a measure of general intelligence, corresponding to approximate IQ bands of <74, 74–81, 82–89, 90–95, 96–104, 105–110, 111–118, 119–126 and >126. This general intelligence variable was used in the analyses in the present study. In the full cohort of men, 49 262 (99.9%) had a score on general intelligence.

Assessment of psychosocial functioning was made through a semi-structured interview administered by a certified psychologist. The overall objective of the interview was to assess the conscript's ability to cope with the psychological requirements of military service and, ultimately, of armed combat. Willingness to assume responsibility, independence, having an outgoing character, persistence, emotional stability, and power of initiative were regarded as the requirements for ‘high ability’ [26]. In the interview, usually lasting between 20 and 30 minutes, the psychologist asked not only about adjustment problems and conflicts, but also about successes, responsibilities taken on, and initiatives shown or experienced, in school, at work, in sports or other leisure activities, and at home [22]. Each conscript's mental energy, stability of emotions, social maturity, and active/passive interests were rated by the psychologist, who then assigned the conscript a summary score between 1 and 9 on psychosocial functioning, a variable constructed to follow a normal distribution. A high ranking on psychosocial functioning could be argued to bear similarities with low neuroticism, high conscientiousness, and high extraversion [22], and would thus be similar to the ‘general factor of personality’ that is found, for example, among traits of the currently-popular five factor model of personality traits [27]. Inter-rater reliability for the assessment of psychosocial functioning was found to be high (r = 0.86) in a test where 30 recorded interviews from 1972/1973 were scored by 30 psychologists [28].

### Measures of socioeconomic status

For the present study, the cohort of conscripts was linked to the Longitudinal Database of Education, Income and Occupation (LOUISE) of 1990–2002, held by Statistics Sweden, in order to obtain information on educational level in 1990 for each study member. In the study, educational level was divided into five categories: ≤9 years of education, 10–11 years, 12–13 years, 14 years, and ≥15 years. This categorization reflects the educational arrangements of the time and roughly corresponds to compulsory level, vocational secondary level, pre-academic secondary level, university degree after 2 years of study, and university degree after 3 or more years of study. The majority of the cohort had completed no more than two years of post-compulsory education by the time of the conscription examination in 1969/70.

Linkage with the National Population and Housing Census of 1990 (response rate >98%) provided information on occupational class. A classification into the following eight classes was conducted by Statistics Sweden: unskilled workers, skilled workers, non-manual employees at lower (assistant), intermediate, or higher level, farmers, self-employed, and those for whom no occupation was reported. In the present study, we used the first five classes, which are hierarchically ordered. The self-employed were allocated to any one of these classes, based on occupational information in the 1990 census, and a number of men who had no reported occupation in the 1990 census were allocated on the basis of occupational information from the corresponding census in 1985. The 1985 and 1990 censuses also provided register-based information on level of income – any taxable income – before the follow-up. The average income of each subject for these years was divided into income quintiles in the present study. The extreme categories represent a number of people within the study population with very high or very low incomes (by Swedish standards in the late 1980's), which means that the differences between these categories and the middle categories are greater than the differences among the three middle categories.

### Measures of mortality

In order to obtain information on mortality, the cohort of conscripts was linked to the National Cause of Death Register 1991–2008, held by the National Board of Health and Welfare. The conscripts were followed with regard to all-cause mortality, and to major cause-specific mortality: cardiovascular disease (CVD) mortality [ICD codes, 9th (390–459), and 10^th^ (I00–I99) revisions], mortality from injuries/violent causes [ICD codes, 9^th^ (800–999) and 10th (V–Y) revisions], cancer mortality [ICD codes, 9th (139–209) and 10th (C) revisions], and alcohol-related mortality [ICD codes, 9th (291, 303) and 10th (F10, K70, K74) revisions].

### Measures of childhood socioeconomic circumstances and somatic diagnosis at conscription

To some extent, lower IQ and psychosocial functioning could reflect poverty and poor health in childhood [29]. Therefore, parental SES, crowded housing in childhood, and having a somatic diagnosis at conscription were treated as possible confounders in the present study. Parental SES refers to the father's occupational position (or that of any other head of household), a classification into seven groups made at Statistics Sweden, and crowded housing refers to ≥2 people per room (kitchen not included). Both variables were based on information obtained by linking the cohort with the National Population and Housing Census of 1960. Somatic diagnosis refers to having any non-psychiatric diagnoses recorded at conscription (ICD-8), excluding some frequent diagnoses unlikely to have an impact on SES attainment or premature death (e.g., refractive error).

### Statistical analyses

For descriptive purposes, we computed mean IQ and psychosocial functioning, with standard deviations, measured at age 18–20 by educational level, occupational class, and level of income measured at ∼39 years of age, along with Spearman's correlation coefficients. We also calculated cumulative incidence of all-cause and cause-specific mortality across levels of education, occupational class, and income, in order to display the SES-related inequalities that are hypothetically explained by intelligence and psychosocial functioning.

Associations of IQ and psychosocial functioning, respectively, with cause-specific and all-cause mortality were estimated using Cox proportional-hazards regression, as implemented in the SAS (version 9.3) PHREG procedure. The increase/decrease in hazard ratios (HR) by lower level of IQ/psychosocial functioning was estimated using variables divided into three groups. Proportionality of hazards was checked with the LIFETEST procedure in SAS (survivor functions).

Associations between SES and premature death were estimated using the relative index of inequality (RII), which is in line with most corresponding previous studies. RII is a widely used regression-based summary measure that takes the size of hierarchical SES groups into account [30]. It is obtained by assigning each SES group a value between 0 (lowest rank) and 1 (highest rank), derived from the group's proportionate size and corresponding to the midpoint of the SES group's range. The mortality rate of the SES groups is then regressed on the RII scores, in the present study using Cox proportional-hazards regression (thus, RII = hazard ratio). For interpretation, a given RII should be seen as the ratio of the mortality between extremes in the lowest-ranking SES group and the highest-ranking SES group [31].

To analyze the extent to which IQ and psychosocial functioning statistically explained the associations between SES and causes of premature death in the cohort, we compared different regression models. The reference model (“Base”) included, in addition to SES in early middle age as predictor and subsequent mortality as outcome, the three following covariates: childhood social class (categorical) and crowded housing (dichotomous), and having a somatic diagnosis in late adolescence (dichotomous); the second model included IQ in addition to these; the third model included, instead, psychosocial functioning as an additional covariate; the fourth model included both IQ and psychosocial functioning as additional covariates. Thus, we sought to minimize confounding by childhood background variables in the estimation of the contributions of IQ and psychosocial functioning; we have previously demonstrated modest correlations between childhood SES and IQ/psychosocial functioning [22].

Percentage attenuations (with 95% confidence intervals) of SES–mortality associations between the base model and other models were calculated in 1000 bootstrap samples. These analyses were conducted with R 2.15.2 statistical software and the ‘boot’ package [32]. The formula for percentage attenuation was (RII_base model_-RII_other model_)/(RII_base model_-1)x100. Incidence rates/differences of all-cause mortality for lowest vs. highest SES groups were calculated and multiplied by percentage attenuations to provide a rough approximation of absolute differences in all-cause mortality possibly accounted for by IQ and psychosocial functioning together (reported in text only).

## Results

There were 1050 out of 49 321 individuals who had died before the start of follow-up, on 1 January 1991. Among the individuals alive at baseline, 428 for whom we lacked reliable information on IQ or psychosocial functioning were excluded; another 804 individuals were excluded due to the absence of information on social class or crowded housing in childhood; and a further 908 were excluded due to the absence of education or income data at baseline. Finally, 3939 individuals who could not be assigned a hierarchically defined SES on the basis of occupation also had to be excluded: men who were farmers and men without occupational information both in 1985 and in 1990. Thus, we were able to follow up 42 192 people with regard to mortality between 1991 and 2008. At the end of the follow-up period, 1971 among those men had died (all causes of mortality). Major specific causes were 498 cases of CVD mortality, 601 cases of cancer mortality, 416 cases of injury mortality, and 148 cases of alcohol-related mortality.

Table 1 shows the mean values (with standard deviations) of IQ and psychosocial functioning per SES category, as measured by level of education, occupational class or level of income, along with the relationships expressed as Spearman's correlation coefficients (unadjusted and adjusted). IQ and psychosocial functioning had a correlation of approximately 0.30. All correlations between IQ/psychosocial functioning and SES variables were positive and statistically significant. IQ had stronger correlations with all indicators of SES in early middle age than did psychosocial functioning, particularly so after mutual adjustment, with the strongest being ρ = 0.52 for IQ (with education), and ρ = 0.18 for psychosocial functioning (with income).

**Table 1:** Averages of intelligence and psychosocial functioning across levels of socioeconomic status.

		IQ	PF
	No. of men	Mean (SD)	Mean (SD)
	42192	5.43 (2.04)^a^	5.10 (1.94)^b^
**Years in education**			
**≥15**	6944	7.15 (1.47)	5.77 (1.98)
**14**	5283	6.50 (1.61)	5.62 (1.91)
**12–13**	7140	6.02 (1.76)	5.43 (1.81)
**10–11**	12227	4.83 (1.76)	4.84 (1.86)
**≤9**	10598	4.05 (1.79)	4.47 (1.85)
		ρ = 0.55***/ρ_adj_ = 0.52***	ρ = 0.25***/ρ_adj_ = 0.10***
**Occupational class**			
**NMH**	8081	6.90 (1.55)	5.81 (1.92)
**NMI**	9209	6.23 (1.73)	5.51 (1.86)
**NML**	4602	5.47 (1.83)	5.20 (1.95)
**SMW**	10481	4.61 (1.80)	4.77 (1.80)
**UMW**	9819	4.32 (1.95)	4.42 (1.88)
		ρ = 0.48***/ρ_adj_ = 0.44***	ρ = 0.26***/ρ_adj_ = 0.14***
**Income quintiles**			
**5^th^**	8961	6.70 (1.67)	5.91 (1.86)
**4^th^**	9088	5.71 (1.87)	5.36 (1.83)
**3^rd^**	9043	5.10 (1.91)	5.00 (1.82)
**2^nd^**	8912	4.67 (1.97)	4.63 (1.86)
**1^st^**	6188	4.73 (2.10)	4.37 (2.00)
		ρ = 0.35***/ρ_adj_ = 0.30***	ρ = 0.27***/ρ_adj_ = 0.18***
**IQxPF**		ρ = 0.30***	

ρ = Spearman's correlation coefficient; ρ_adj_ = partial Spearman's, i.e. adjusted for PF/IQ; *** = p<0.001; NMH = Non-manual workers, higher level; NMI = Non-manual workers, intermediate level; NML = Non-manual workers, lower level; SMW = Skilled manual workers; UMW = Unskilled manual workers; ^a^ Skewness = −0.191 (p<0.001); ^b^ Skewness = −0.073 (p<0.001). Mean and standard deviation (SD) of intelligence (IQ) and psychosocial functioning (PF) across levels of SES indicators;

Table 2 shows the associations of IQ and psychosocial functioning, measured at 18–20 years of age, with cause-specific and all-cause mortality between 40 and 57 years of age. Lower scores of both IQ and psychosocial functioning were found to be associated with rather similar hazard ratios for all-cause mortality, CVD mortality, injury mortality, and alcohol-related mortality. A score of 1–3 (vs. the reference category, 7–9) of IQ or psychosocial functioning was associated with almost doubled hazard ratios of all-cause and CVD mortality, and 2,5 times increased hazard ratios of mortality related to injury or alcohol. Hazard ratios for cancer mortality were, on the other hand, not much increased even for the lowest scores of IQ or psychosocial functioning: confidence interval for IQ was not different from 1.0, and confidence interval for psychosocial functioning was close to 1.0. Lung cancer deaths were increased among men with low scores of IQ/psychosocial functioning, but there were only 102 cases occurring (not shown in the table): IQ, 1–3: HR = 2.16 (1.11–4.19); psychosocial functioning, 1–3: HR = 1.87 (1.02–3.42).

**Table 2:** Associations of intelligence and psychosocial functioning with all-cause and cause-specific mortality from 40 to 57 years of age.

	All-cause (1971)		CVD (498)		Cancer (610)		Injury (416)		Alcohol (148)	
	*HR*	*(95% CI)*	*HR*	*(95% CI)*	*HR*	*(95% CI)*	*HR*	*(95% CI)*	*HR*	*(95% CI)*
**IQ**										
**7–9**	1.00		1.00		1.00		1.00		1.00	
**4–6**	1.36	1.21–1.54	1.34	1.06–1.71	1.12	0.92–1.36	1.57	1.18–2.07	2.23	1.35–3.69
**1–3**	1.87	1.63–2.15	1.99	1.52–2.61	1.17	0.91–1.49	2.58	1.90–3.50	2.48	1.40–4.38
**PF**										
**7–9**	1.00		1.00		1.00		1.00		1.00	
**4–6**	1.26	1.19–1.42	1.42	1.12–1.79	1.07	0.87–1.30	1.33	1.02–1.75	1.46	0.92–2.33
**1–3**	1.91	1.68–2.17	1.73	1.33–2.25	1.37	1.10–1.72	2.39	1.81–3.16	2.64	1.63–4.26

% confidence intervals, HR (95% CI); intelligence (IQ) and psychosocial functioning (PF) are stanine variables divided into 1–3, 4–6, and 7–9 (i.e. high = reference). Cox proportional-hazards regressions yielding hazard ratios and 95

Mortality before 40 years of age, when attained SES was measured, is not included in Table 2. However, the associations between IQ/psychosocial functioning and this mortality (>1000 cases) were of at least the same magnitude.

**Figure 1:**
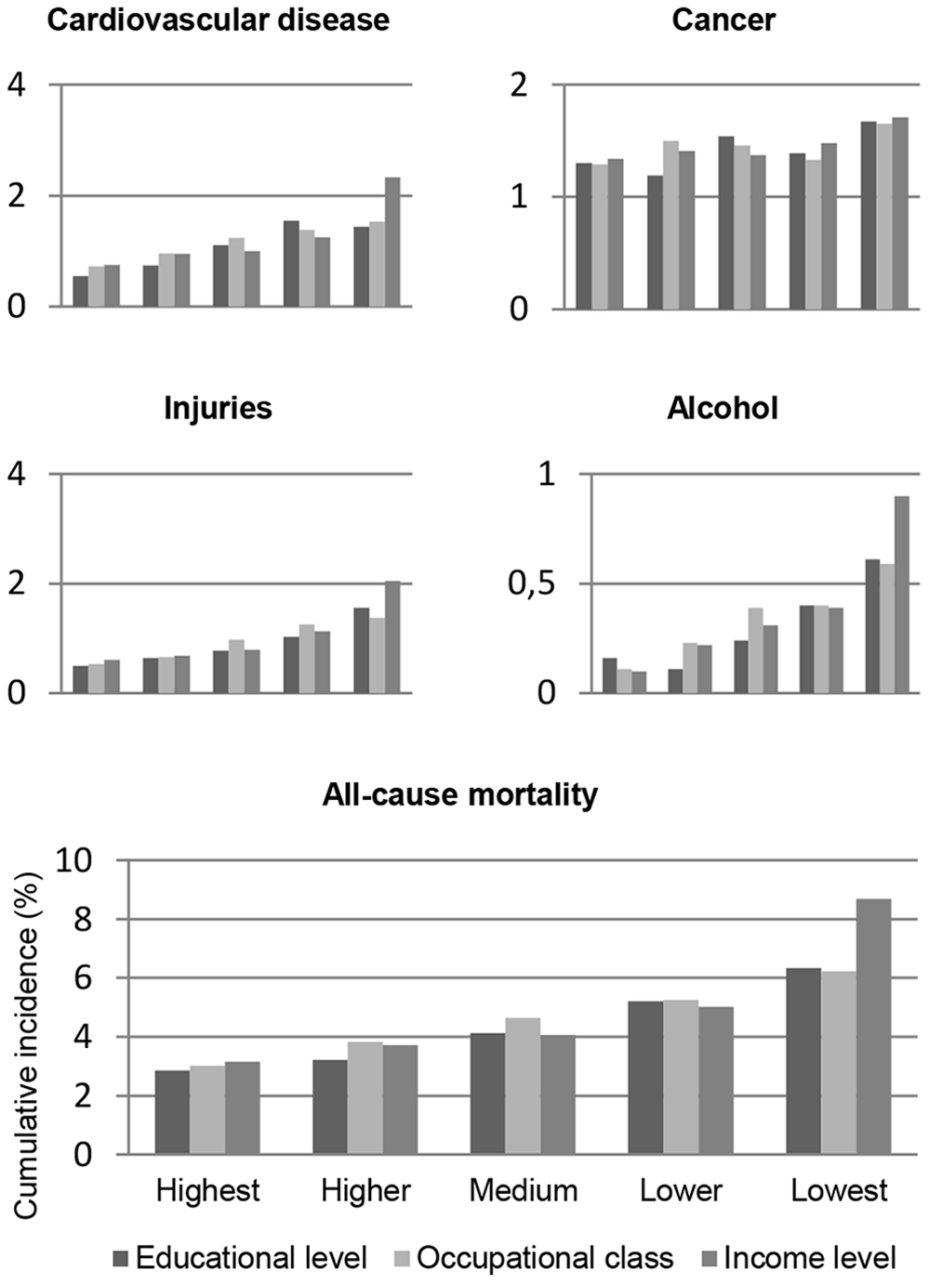
Socioeconomic inequalities in causes of premature death. Cumulative incidence (%) of cause-specific and all-cause mortality by socioeconomic status among Swedish men during the follow-up from 40 to 57 years of age, with socioeconomic status measured by level of education, occupational class, and level of income. Those are socioeconomic inequalities in premature death possibly explained by personality and intelligence differences to some extent.

Figure 1 shows the associations between the SES indicators (education, occupational class, income), measured at about 39 years of age, and the mortality subsequently followed in the cohort. It is seen that lower levels of education, occupational class and income were all associated with mortality, in terms of cumulative incidence, from CVD, injuries, and alcohol-related causes, and also with overall mortality. For SES as measured by income, these increases in mortality were particularly marked in the lowest income quintile. Mortality due to cancer, on the other hand, was more evenly distributed across SES groups, regardless of SES measure.

Table 3 demonstrates how adjustments for IQ and psychosocial functioning attenuated the associations between SES indicators and mortality from the different causes. IQ and psychosocial functioning were added as covariates to the base model already adjusted for indicators of social circumstances in childhood and having a somatic diagnosis at conscription (which had limited effects on the crude SES-mortality associations; see supporting information in Table S1).

**Table 3:** SES-related inequalities in cause-specific and all-cause mortality, adjusted for intelligence and psychosocial functioning.

	Measures of socioeconomic status								
	Education			Occupational class			Income		
	*RII*	*95%CI*	*% attenuation*	*RII*	*95%CI*	*% attenuation*	*RII*	*95%CI*	*% attenuation*
Models									
**All-cause**									
Base^a^	2.77	2.33–3.30		2.50	2.10–2.97		3.36	2.85–3.96	
+ IQ	2.33	1.92–2.84	*25% (13*–*37%)*	2.07	1.71–2.50	*29% (18*–*40%)*	3.00	2.53–3.56	*15% (9*–*21%)*
+ PF	2.38	1.99–2.84	*22% (17*–*27%)*	2.11	1.77–2.51	*26% (20*–*32%)*	2.89	2.44–3.42	*20% (14*–*25%)*
+ IQ&PF	2.20	1.80–2.68	*32% (20*–*45%)*	1.89	1.56–2.29	*41% (29*–*52%)*	2.72	2.29–3.24	*27% (20*–*34%)*
**CVD**									
Base^a^	2.75	1.95–3.89		2.40	1.70–3.39		3.67	2.64–5.10	
+ IQ	2.06	1.39–3.05	*39% (16*–*62%)*	1.79	1.23–2.62	*44% (21*–*66%)*	3.14	2.22–4.43	*20% (9*–*31%)*
+ PF	2.43	1.70–3.45	*18% (8*–*28%)*	2.09	1.47–2.97	*22% (9*–*35%)*	3.28	2.34–4.61	*15% (5*–*24%)*
+ IQ&PF	1.97	1.33–2.92	*45% (20*–*69%)*	1.68	1.15–2.47	*51% (24*–*76%)*	2.95	2.08–4.19	*27% (14*–*40%)*
**Cancer**									
Base^a^	1.54	1.14–2.09		1.34	0.99–1.81		1.40	1.03–1.91	
+ IQ	1.70	1.20–2.40	*	1.38	0.99–1.93	*	1.41	1.04–1.91	*
+ PF	1.42	1.04–1.94	*	1.22	0.90–1.66	*	1.27	0.94–1.70	*
+ IQ&PF	1.63	1.15–2.30	*	1.30	0.94–1.82	*	1.31	0.96–1.78	*
**Injury**									
Base^a^	4.37	2.97–6.43		3.78	2.57–5.55		4.80	3.34–6.89	
+ IQ	3.02	1.96–4.67	*40% (22*–*59%)*	2.61	1.71–3.99	*42% (25*–*59%)*	3.83	2.62–5.60	*26% (14*–*36%)*
+ PF	3.55	2.40–5.26	*24% (15*–*33%)*	3.00	2.02–4.45	*28% (19*–*38%)*	3.89	2.68–5.65	*24% (14*–*34%)*
+ IQ&PF	2.81	1.81–4.34	*46% (28*–*65%)*	2.34	1.53–3.58	*52% (35*–*68%)*	3.37	2.29–4.96	*38% (24*–*50%)*
**Alcohol**									
Base^a^	6.58	3.35–12.93		5.99	3.03–11.84		11.44	5.95–22.01	
+ IQ	5.71	2.70–12.10	*16% (−16*–*47%)*	4.99	2.39–10.41	*20% (−6*–*46%)*	10.32	5.26–20.27	*11% (−4*–*26%)*
+ PF	5.20	2.62–10.33	*25% (12*–*38%)*	4.61	2.30–9.24	*28% (11*–*43%)*	9.24	4.72–18.08	*21% (7*–*36%)*
+ IQ&PF	5.21	2.46–11.05	*25% (−5*–*56%)*	4.33	2.07–9.05	*33% (8*–*59%)*	8.88	4.48–17.59	*25% (8*–*41%)*

% confidence interval (95% CI), estimated using Cox proportional-hazards regression; % attenuation = (RII_Crude_−RII_Adjusted_)/(RII_Crude_−1)x100, i.e., percentage change in RII between base and adjusted model; *Not reported due to high uncertainty (p-values>0.45); IQ = intelligence; PF = psychosocial functioning; ^a^ Adjusted for childhood social class and crowded housing, and having a somatic diagnosis recorded at conscription examination. Relative index of inequality (RII) with 95

For all-cause mortality, IQ and psychosocial functioning together accounted for 32% (20–45%) of SES inequality as measured by educational differences, 41% (confidence interval: 29–52%) of inequality as measured by differences in occupational class, and 27% (20–34%) of inequality as measured by income level, in terms of % attenuation. Roughly calculated (not shown in the table), these percentages could translate into 63 cases out of a 198 cases difference per 100,000 person-years/75 cases out of a 183 cases difference per 100,000 person-years/89 cases out of a 328 cases difference per 100,000 person-years of all-cause mortality inequalities by lowest vs. highest educational level/occupational class/income level.

For mortality from CVD, 45% (20–69%) and 51% (24–76%) of the inequality as measured by educational level or occupational class was statistically explained by differences in IQ and psychosocial functioning together (Table 3). However, less was explained when the SES difference was measured by level of income. In the separate models, IQ-adjustment attenuated the inequalities in CVD mortality more than did psychosocial functioning.

The small inequalities in cancer mortality did not allow for calculations of percentage attenuation with any reasonable certainty (p-values>0.45). Adjustment for IQ increased the RIIs, which may be due to a higher number of cancer deaths in the upper part of the IQ range (not shown in the tables), while adjustment for psychosocial functioning decreased the RIIs, regardless of the SES measure used.

For death due to injuries, 52% (35–68%) of the inequalities by occupational class were explained by differences in IQ and psychosocial functioning, with IQ making a larger contribution in terms of % attenuation (Table 3). For the inequality in alcohol-related mortality by occupational class, IQ contributed less than psychosocial functioning and their joint contribution—33% (8–59%)—was smaller than for injury mortality. Again, the statistical contributions were somewhat smaller when inequality was measured by income level, which was seen most clearly for the contribution of IQ.

Throughout Table 3, the attenuating effect of adjusting for both IQ and psychosocial functioning was less than the sum of the effects of adjusting for IQ or psychosocial functioning alone, probably accounted for by their correlation of 0.30. Taken as a whole, the table shows substantial attenuation of SES associations and, at the same time, that SES-related inequalities in all-cause and cause-specific mortality remained after IQ and psychosocial functioning adjustments.

## Discussion

The present study shows that lower psychosocial functioning and IQ both contribute to accounting for SES-related inequalities in premature death among middle-aged Swedish men. The estimated inequalities in all-cause mortality by education and occupational class were ∼30–40% smaller after adjustments for these individual-difference measures. The inequalities in cardiovascular and injury mortality were ∼45–50% smaller after the same adjustments, and the inequalities in alcohol-related mortality were 25–33% smaller. The small SES-related inequalities in cancer mortality were not attenuated by adjustment for IQ.

### Methodological considerations

The present study was based on a large population, highly representative of men born around 1950 in Sweden. Only a very small proportion among Swedish men, aged 18–19 years in most cases, was exempted from the 1969/70 conscription examinations. The study used prospectively measured data on childhood background factors, IQ and psychosocial functioning in late adolescence, SES in early middle age, and mortality between 40 and 57 years of age, obtained from sources with minimal loss of information. Earlier studies have to a greater extent been based on cross-sectional measurements of IQ, personality characteristics, and SES [7], [10], [16], [17], and have typically been of limited size [33]. Self-rating problems were reduced: IQ was measured through a comprehensive multidimensional test and psychosocial functioning was rated on the basis of a 20–30 minutes long interview with a psychologist. Psychosocial functioning cannot be straight-forwardly compared with personality inventories such as the NEO-PI (“Big Five”) [34] since it is a one-dimensional summary measure. However, it should account for significant individual variation in psychological characteristics like level of activity, power of initiative, independence, and emotional stability, which we argue have similarities with high extraversion, high conscientiousness, and low neuroticism combining to form what is called the general factor of personality [27]. Estimated correlations between this and general intelligence have been rather similar to the correlation between psychosocial functioning and IQ (ρ = 0.30) found in the present study [27]. At the same time, it is possible that adjustments for psychosocial functioning in our analyses captured some effects of IQ.

Women could not be studied, since a corresponding source of information on IQ and psychosocial functioning did not exist for them. Whether or not associations between IQ/psychosocial functioning, SES in adulthood and mortality in women would be similar to the ones found in the present study is uncertain [5], [9].

### Comparison with previous studies

A number of previous studies have investigated the extent to which socioeconomic inequalities in mortality may be confounded by intelligence differences. Research results – and interpretations – have been conflicting. Analyses from the Whitehall II study showed that IQ statistically explained over 20% of socioeconomic differences in CHD and mental functioning, and 30–40% of socioeconomic differences in physical functioning and self-rated health. However, associations between IQ and a majority of the health outcomes were found to be statistically non-significant after adjusting for SES differences and, therefore, the authors rejected the hypothesis that intelligence differences are an important explanation of SES-related inequalities in health [7]. Furthermore, analyses of the U.S. National Longitudinal Survey of Youth study suggested that IQ could not explain the association between education/income and mortality before the age of 50 [8], and a similar conclusion was drawn from a study in Malmö, Sweden [9]. Analyses of the West of Scotland Twenty-07 study, on the other hand, showed a stronger statistical explanatory power of IQ; for example, the associations found between education/social class and mortality/CHD mortality completely disappeared after adjusting for IQ, even though the IQ-adjusted associations between SES indicators and other health outcomes, which were “softer”, generally remained statistically significant and showed less attenuation in effect sizes [10]. Analyses of the Vietnam Experience study also showed substantial statistical explanatory power of IQ with regard to socioeconomic differences in total and CVD mortality; IQ showed greater explanatory power than did traditional CVD risk factors [12]. Furthermore, analyses of data linked between the Scottish Mental Survey of 1932 and the Midspan Studies [11] indicated the importance of IQ in this context, as did a study of more than 1 million relatively young Swedish men with regard to inequalities in mortality due to injuries [13]. The analyses in the present study showed that IQ could statistically explain roughly 45–50% of educational or occupational class inequalities in premature death due to injuries and CVD, rather similar findings to those of earlier studies [7], [12], [13]. The analyses also showed that IQ accounted for somewhat less of the SES-related inequalities in alcohol-related mortality. Finally, IQ was shown not to explain the small SES-related inequalities observed in cancer mortality, and, partly because of this, the contribution of IQ to inequalities in all-cause mortality was less, about 25%.

In the present study, analyses showed that psychosocial functioning also could account for about 25% of the inequalities in all-cause mortality. Further, it explained roughly 20% of the educational and occupational class inequalities in CVD mortality, and about 25–30% of the inequalities in injury and alcohol-related mortality. The potential importance of personality differences for SES-related inequalities in health was previously indicated in two studies (lacking information on IQ, however). The French GAZEL study showed that some specific measures of personality could together statistically explain about 30% of the associations between SES indicators and total mortality among middle-aged men; and about 40% of the associations with CVD mortality in men was explained by a measure of “coronary prone personality” [16]. In the MIDUS cohort [17], analyses showed that about 20% of the SES gradient in total mortality was statistically explained by differences in general personality traits from the Five Factor Model [34]. In the Vietnam Experience study, however, the neuroticism dimension of personality appeared unrelated to income inequalities in mortality [18].

The analyses in the present study showed that psychosocial functioning and IQ both contributed partly to explaining socioeconomic differences in major causes of mortality, and that their contributions correlated to some extent. Previous studies have not been able to examine this issue, and more studies are needed to establish the extent to which intelligence, psychosocial functioning and personality dimensions overlap in terms of their explanatory contributions to socioeconomic differences in mortality. Correlations between intelligence and aspects of personality are known in the literature [19].

In the present study, we found that the statistical explanations by IQ of social inequalities in mortality were consistently smaller when SES was measured by income level than when measured by level of education or occupational class, which is in line with earlier studies [7], [10], [11], [12]. This was not seen for our measure related to personality, in agreement with the one previous study with which a comparison can be made [16]. Smaller contributions from IQ when SES was measured by income resulted, overall, in less explanation of income inequalities in mortality than when it was measured by education or occupational class. This may possibly be because income, as compared to the other measures of SES, is more variable over time in individuals and captures change, and, furthermore, that it is to a greater extent affected by circumstances in present time, such as problems with health and employment [23]. Psychosocial functioning may be relatively more predictive of changing circumstances like these than of SES attainment earlier in adulthood [22], [26].

### Interpretation

Higher scores on psychosocial functioning at 18–20 years of age, and even more on IQ, were found to be associated with higher SES attained in early middle age. Both IQ and psychosocial functioning were also related to all-cause and cause-specific mortality from 40 to 57 years of age. When this was taken into account, the associations between SES and major causes of death were attenuated. This was demonstrated in models where childhood background variables had already been accounted for. Thus, we cannot reject the hypothesis that observed associations between lower attained SES and higher risk of mortality, illustrated in Figure 1, are in part accounted for by individual psychological differences, although the idea that intelligence is the fundamental cause of social class inequalities in health [6] is only partly supported; it might be better characterized as one of the possible causes. Reaching this conclusion on the basis of a Swedish population is, by the way, no paradox; welfare states with egalitarian (e.g. educational) policies provide an environment in which intrinsic resources more than social background may serve as a link to higher attained SES [35], [36]. Within such societies, egalitarian policies affecting children may have reduced the extent to which social disadvantage can account for intelligence and personality differences [29].

Individual psychological differences might reflect varying ability among individuals to deal with long-term risks of disease and death. With regard to intelligence, Gottfredson and Deary [20] have argued that its relationship to health may mainly be due to efficient self-care and safer behaviors among individuals higher in intelligence. Negative health behaviors, such as tobacco smoking, have been shown to be potentially important factors explaining associations between individual characteristics, SES in adulthood, and health outcomes [25], [37]. Over the life course, significant individual differences may thus drive accumulation of advantage/disadvantage in terms of both SES and health [35].

Of the SES-related inequalities in total mortality, around 60–70% remained unexplained by IQ and psychosocial functioning as measured in the present study. In other words, major parts of the inequalities seen in Figure 1 could have explanations unrelated to individual differences in personality and intelligence.

In conclusion, personality characteristics and intelligence might both contribute to cause SES-related inequalities in premature death, but the magnitude of their contributions likely varies with measure of socioeconomic status and cause of death. Both personality characteristics and intelligence should be considered in future studies.

## Supporting Information

Table S1 **SES-related inequalities in cause-specific and all-cause mortality, adjusted for intelligence and psychosocial functioning; crude models vs. basic adjustment.**
(DOCX)

## References

[1] Deary IJ, Weiss A, Batty GD (2010) Intelligence and personality as predictors of illness and death: how researchers in differential psychology and chronic disease epidemiology are collaborating to understand and address health inequalities. Psychol Sci Public Interest 11: 53–79. 26168413 10.1177/1529100610387081

[2] Ozer DJ, Benet-Martinez V (2006) Personality and the prediction of consequential outcomes. Annu Rev Psychol 57: 401–421. 16318601 10.1146/annurev.psych.57.102904.190127

[3] Roberts BW, Kuncel NR, Shiner R, Caspi A, Goldberg LR (2007) The Power of Personality The Comparative Validity of Personality Traits, Socioeconomic Status, and Cognitive Ability for Predicting Important Life Outcomes. Perspect Psychol Sci 2: 313–345. 26151971 10.1111/j.1745-6916.2007.00047.x PMC4499872

[4] Strenze T (2007) Intelligence and socioeconomic success: A meta-analytic review of longitudinal research. Intelligence 35: 401–426.

[5] Calvin CM, Deary IJ, Fenton C, Roberts BA, Der G, et al (2011) Intelligence in youth and all-cause-mortality: systematic review with meta-analysis. International Journal of Epidemiology 40: 626–644. 21037248 10.1093/ije/dyq190 PMC3147066

[6] Gottfredson LS (2004) Intelligence: Is it the epidemiologists' elusive “Fundamental cause” of social class inequalities in health? Journal of Personality and Social Psychology 86: 174–199. 14717635 10.1037/0022-3514.86.1.174

[7] Singh-Manoux A, Ferrie JE, Lynch JW, Marmot M (2005) The role of cognitive ability (intelligence) in explaining the association between socioeconomic position and health: Evidence from the Whitehall II prospective cohort study. American Journal of Epidemiology 161: 831–839. 15840615 10.1093/aje/kwi109

[8] Jokela M, Elovainio M, Singh-Manoux A, Kivimaki M (2009) IQ, Socioeconomic Status, and Early Death: The US National Longitudinal Survey of Youth. Psychosomatic Medicine 71: 322–328. 19251867 10.1097/PSY.0b013e31819b69f6 PMC2851186

[9] Lager A, Bremberg S, Vagero D (2009) The association of early IQ and education with mortality: 65 year longitudinal study in Malmo, Sweden. British Medical Journal 339. 10.1136/bmj.b5282 PMC2792333 20008007

[10] Batty GD, Der G, Macintyre S, Deary IJ (2006) Does IQ explain socioeconomic inequalities in health? Evidence from a population based cohort study in the west of Scotland. British Medical Journal 332: 580–583. 16452104 10.1136/bmj.38723.660637.AE PMC1397779

[11] Hart CL, Taylor MD, Smith GD, Whalley LJ, Starr JM, et al (2003) Childhood IQ, social class, deprivation, and their relationships with mortality and morbidity risk in later life: Prospective observational study linking the Scottish Mental Survey 1932 and the Midspan studies. Psychosomatic Medicine 65: 877–883. 14508035 10.1097/01.psy.0000088584.82822.86

[12] Batty GD, Shipley MJ, Dundas R, Macintyre S, Der G, et al (2009) Does IQ explain socio-economic differentials in total and cardiovascular disease mortality? Comparison with the explanatory power of traditional cardiovascular disease risk factors in the Vietnam Experience Study. European Heart Journal 30: 1903–1909. 19602715 10.1093/eurheartj/ehp254 PMC2719700

[13] Batty GD, Gale CR, Tynelius P, Deary IJ, Rasmussen F (2009) IQ in Early Adulthood, Socioeconomic Position, and Unintentional Injury Mortality by Middle Age: A Cohort Study of More Than 1 Million Swedish Men. American Journal of Epidemiology 169: 606–615. 19147741 10.1093/aje/kwn381 PMC2640161

[14] Krueger RF, Caspi A, Moffitt TE (2000) Epidemiological personology: The unifying role of personality in population-based research on problem behaviors. Journal of Personality 68: 967–998. 11130741 10.1111/1467-6494.00123

[15] Matthews G, Deary I, Whiteman M (2003) Personality Traits. Cambridge, UK: Cambridge University Press.

[16] Nabi H, Kivimaki M, Marmot MG, Ferrie J, Zins M, et al (2008) Does personality explain social inequalities in mortality? The French GAZEL cohort study. International Journal of Epidemiology 37: 591–602. 18276626 10.1093/ije/dyn021 PMC2650255

[17] Chapman BP, Fiscella K, Kawachi I, Duberstein PR (2010) Personality, Socioeconomic Status, and All-Cause Mortality in the United States. American Journal of Epidemiology 171: 83–92. 19965888 10.1093/aje/kwp323 PMC2800299

[18] Weiss A, Gale CR, Batty GD, Deary IJ (2009) Emotionally Stable, Intelligent Men Live Longer: The Vietnam Experience Study Cohort. Psychosomatic Medicine 71: 385–394. 19251871 10.1097/PSY.0b013e318198de78

[19] Borghans L, Golsteyn BHH, Heckman J, Humphries JE (2011) Identification problems in personality psychology. Personality and Individual Differences 51: 315–320. 21731170 10.1016/j.paid.2011.03.029 PMC3126096

[20] Gottfredson LS, Deary IJ (2004) Intelligence Predicts Health and Longevity, but Why? Current Directions in Psychological Science 13: 1–4.

[21] Smith TW (2006) Personality as Risk and Resilience in Physical Health. Current Directions in Psychological Science 15: 227–231.

[22] Sorjonen K, Hemmingsson T, Lundin A, Falkstedt D, Melin B (2012) Intelligence, socioeconomic background, emotional capacity, and level of education as predictors of attained socioeconomic position in a cohort of Swedish men. Intelligence 40: 269–277.

[23] Galobardes B, Lynch J, Smith GD (2007) Measuring socioeconomic position in health research. British Medical Bulletin 81–82: 21–37. 10.1093/bmb/ldm001 17284541

[24] Hemmingsson T, Melin B, Allebeck P, Lundberg I (2006) The association between cognitive ability measured at ages 18–20 and mortality during 30 years of follow-up - a prospective observational study among Swedish males born 1949–51. International Journal of Epidemiology 35: 665–670. 16446349 10.1093/ije/dyi321

[25] Hemmingsson T, Melin B, Allebeck P, Lundberg I (2009) Cognitive ability in adolescence and mortality in middle age: a prospective life course study. Journal of Epidemiology and Community Health 63: 697–702. 19574248 10.1136/jech.2008.079160

[26] Lindqvist E, Vestman R (2011) The Labor Market Returns to Cognitive and Noncognitive Ability: Evidence from the Swedish Enlistment. American Economic Journal-Applied Economics 3: 101–128.

[27] Just C (2011) A review of literature on the general factor of personality. Personality and Individual Differences 50: 765–771.

[28] Lilieblad B, Ståhlberg B (1977) Reliability of the psychological assessments at conscription. FOA-rapport C55011-07. Stockholm: Armed forces research department, Sweden.

[29] Heckman JJ (2007) The economics, technology, and neuroscience of human capability formation. Proceedings of the National Academy of Sciences of the United States of America 104: 13250–13255. 17686985 10.1073/pnas.0701362104 PMC1948899

[30] Mackenbach JP, Kunst AE (1997) Measuring the magnitude of socio-economic inequalities in health: An overview of available measures illustrated with two examples from Europe. Social Science & Medicine 44: 757–771. 9080560 10.1016/s0277-9536(96)00073-1

[31] Hayes LJ, Berry G (2002) Sampling variability of the Kunst-Mackenbach relative index of inequality. Journal of Epidemiology and Community Health 56: 762–765. 12239202 10.1136/jech.56.10.762 PMC1732018

[32] Canty A, Ripley B (2012) boot: Bootstrap R (S-Plus) functions: R package version 1.3–7.

[33] Gallacher J (2008) Commentary: Personality and health inequality: inconclusive evidence for an indirect hypothesis. International Journal of Epidemiology 37: 602–603. 18388151 10.1093/ije/dyn062

[34] Costa PT, McCrae RR (1997) Stability and change in personality assessment: The Revised NEO Personality Inventory in the year 2000. Journal of Personality Assessment 68: 86–94. 9018844 10.1207/s15327752jpa6801_7

[35] Mackenbach JP (2005) Genetics and health inequalities: hypotheses and controversies. Journal of Epidemiology and Community Health 59: 268–273. 15767378 10.1136/jech.2004.026807 PMC1733045

[36] Mackenbach JP (2012) The persistence of health inequalities in modern welfare states: The explanation of a paradox. Social Science & Medicine 75: 761–769. 22475407 10.1016/j.socscimed.2012.02.031

[37] Pulkki L, Kivimaki M, Keltikangas-Jarvinen L, Elovainio M, Leino M, et al (2003) Contribution of adolescent and early adult personality to the inverse association between education and cardiovascular risk behaviours: prospective population-based cohort study. International Journal of Epidemiology 32: 968–975. 14681258 10.1093/ije/dyg097

