# Supplementary material for: Psychosocial Functioning and Intelligence Both Partly Explain Socioeconomic Inequalities in Premature Death. A Population-Based Male Cohort Study
Source: PLoS One. 2013 Dec 11;8(12):e82031. doi: 10.1371/journal.pone.0082031 (PMC3859588; doi:10.1371/journal.pone.0082031)
Supplement: Table S1 — SES-related inequalities in cause-specific and all-cause mortality, adjusted for intelligence and psychosocial functioning; crude models vs. basic adjustment. (DOCX) [file pone.0082031.s001.docx]

| **Table S1. SES-related inequalities in cause-specific and all-cause mortality, adjusted for intelligence and psychosocial functioning; crude models vs. basic adjustment*** | | | | | | |
| --- | --- | --- | --- | --- | --- | --- |
|  | **Measures of socioeconomic status** | | | | | |
|  | **Level of education** | | **Occupational class** | | **Level of income** | |
|  | *RII* | *95%CI* | *RII* | *95%CI* | *RII* | *95%CI* |
| Models |  |  |  |  |  |  |
| **Cardiovascular disease** | | |  |  |  |  |
| Crude | 2.88 | 2.08-3.99 | 2.55 | 1.84-3.54 | 3.83 | 2.77-5.28 |
| Base* | 2.75 | 1.95-3.89 | 2.40 | 1.70-3.39 | 3.67 | 2.64-5.10 |
| **Cancer** | | |  |  |  |  |
| Crude | 1.37 | 1.03-1.82 | 1.23 | 0.92-1.63 | 1.32 | 0.99-1.75 |
| Base* | 1.54 | 1.14-2.09 | 1.34 | 0.99-1.81 | 1.40 | 1.05-1.86 |
| **Injury** | | |  |  |  |  |
| Crude | 4.04 | 2.80-5.81 | 3.58 | 2.47-5.17 | 4.76 | 3.33-6.81 |
| Base* | 4.37 | 2.97-6.43 | 3.78 | 2.57-5.55 | 4.80 | 3.34-6.89 |
| **Alcohol** | | |  |  |  |  |
| Crude | 7.01 | 3.67-13.31 | 6.49 | 3.37-12.50 | 11.98 | 6.29-22.82 |
| Base* | 6.58 | 3.35-12.93 | 5.99 | 3.03-11.84 | 11.44 | 5.95-22.01 |
| **All-cause** | | |  |  |  |  |
| Crude | 2.71 | 2.31-3.19 | 2.49 | 2.11-2.93 | 3.38 | 2.88-3.97 |
| Base* | 2.77 | 2.33-3.30 | 2.50 | 2.10-2.97 | 3.36 | 2.85-3.96 |
| Relative index of inequality (RII) with 95% confidence interval (95% CI), estimated with Cox proportional-hazards regressions; IQ=intelligence; PF=psychosocial functioning; *=adjusted for childhood social class and crowded housing, and having a somatic diagnosis recorded at the conscription examination; | | | | | | |
